# Anterior synechiae after penetrating keratoplasty in infants and children with Peters’ anomaly

**DOI:** 10.1186/s12886-022-02473-0

**Published:** 2022-06-09

**Authors:** Yujing Yang, Jun Xiang, Jianjiang Xu

**Affiliations:** 1 Department of Ophthalmology and Visual Science, Eye and ENT Hospital of Fudan University, 83 Fenyang Road, Shanghai, 200031 China; 2 Key Laboratory of Myopia, National Health Commission, Shanghai, China; 3 Shanghai Key Laboratory of Visual Impairment and Restoration, Shanghai, China

**Keywords:** Peters’ anomaly, Congenital corneal opacity, Penetrating keratoplasty, Optical coherence tomography

## Abstract

**Background:**

To evaluate anterior synechiae after penetrating keratoplasty (PK) in patients with Peters’ anomaly using anterior segment optical coherence tomography (OCT).

**Methods:**

A retrospective cross-sectional study was performed. The medical records of patients diagnosed with Peters’ anomaly who underwent PK between 2013 and 2018 were reviewed. In addition to basic ophthalmic examinations, images of anterior segment structures were obtained via spectral-domain OCT at baseline and during the postoperative follow-up period. The profiles of postoperative anterior synechiae and multiple potential risk factors were analyzed.

**Results:**

Seventy-one eyes of 58 patients, aged 5 to 23 months, were included. Various extent of postoperative anterior synechiae was observed in 59 eyes (83.1%). OCT findings revealed graft-host junction synechiae, peripheral anterior synechiae, and a combination of both. Disease severity and malposition of the internal graft-host junction were significantly associated with the formation of postoperative synechiae. Multivariate regression analysis found that preexisting iridocorneal adhesion [odds ratio (OR) = 16.639, 95% confidence interval (CI) 1.494–185.294, *p* = 0.022] was positively correlated with postoperative anterior synechiae, whereas anterior chamber depth (OR = 0.009, 95% CI 0.000–0.360, *p* = 0.012) and graft size (OR = 0.016, 95% CI 0.000–0.529, *p* = 0.020) were negatively correlated with postoperative synechiae. In addition, quadrants of preexisting iridocorneal adhesion and width of the host corneal bed were identified as risk factors for increased postoperative anterior synechiae.

**Conclusions:**

Anterior synechiae following PK is a relatively common occurrence in Peters’ anomaly patients and is found to be associated with preexisting iridocorneal adhesion, a shallow anterior chamber, small graft size, graft-host junction malposition, and graft closer to the corneal limbus. These data indicate the need for careful consideration when performing PK on these patients.

**Supplementary Information:**

The online version contains supplementary material available at 10.1186/s12886-022-02473-0.

## Introduction

Peters’ anomaly is a rare, congenital ocular malformation characterized by central corneal opacity with corresponding defects in the posterior stroma, Descemet's membrane, and endothelium [1, 2]. Typically, iris strands extend from the collarette to the periphery of the corneal leukoma [3, 4]. Peters’ anomaly is not a homogenous disease; cases vary from mild to severe. Co-existing glaucoma was observed in 50–70% of patients [2, 5, 6]. The degree of visual deprivation depends on the size, location, and density of the opacity. When the corneal opacity obstructs the visual axis, penetrating keratoplasty (PK) may be the necessary first step in preventing irreversible loss of visual function. Pediatric keratoplasty is a challenging and demanding procedure because of the technical difficulties encountered while performing the surgery, the high rate of complications, and the threat of amblyopia. PK in pediatric patients with Peters’ anomaly has been reported to have poor surgical outcomes. Factors associated with graft failure included preoperative vascularization, graft size, concomitant lensectomy/vitrectomy, and postoperative complications, especially allograft rejection and glaucoma. A better understanding of intra- and postoperative problems has enabled us to achieve a higher level of anatomical success.

Anterior synechiae (AS) is a common postoperative complication in younger patients who undergo PK due to low scleral rigidity, increased fibrin reaction, and positive vitreous pressure. In adults, previous studies have indicated that AS formation after PK may induce secondary glaucoma and immunological graft rejection, and further lead to graft failure or poor visual prognosis [7–10]. However, little is known about the features, the associated factors, and the potential influences of postoperative AS in pediatric patients with Peters’ anomaly. The ophthalmic examination of infants and children is often challenging owing to poor cooperation. Due to varying degrees of peripheral corneal opacification and postoperative corneal edema, AS could not be properly seen with a slit-lamp or operative microscope. As a fast, high-resolution, and noninvasive imaging technique, anterior segment optical coherence tomography (OCT) has proven to be valuable in pre- and intraoperative examinations of children with congenital corneal opacities [11–13]. The information obtained in this way could be useful in clarifying diagnoses, categorizing Peters’ anomaly, and guiding surgical intervention [11–13].

The purpose of this study was to evaluate the corneal grafts and AS after PK in patients with Peters’ anomaly using anterior segment OCT.

## Materials and methods

### Participants

This study was approved by the Institutional Review Board of Eye and ENT Hospital of Fudan University. Written informed consent was obtained from all patients and their legal guardians. Informed consent was needed to perform the data collection and interpretation, as well as to apply any health care intervention. Data were gathered by a retrospective review of medical records, operative reports, and pathology reports of pediatric patients who were diagnosed with Peters’ anomaly and underwent PK at this hospital from December 2013 to April 2018. Ophthalmic evaluations performed at the initial presentation included slit-lamp examination of the anterior segment, intraocular pressure (IOP) measurement (Tono-Pen XL; Reichert Technologies, DePew, New York, USA), and indirect ophthalmoscopy of the posterior segment when feasible, or B-scan ultrasonography if the fundus was invisible because of corneal opacity. The clinical diagnosis was histopathologically confirmed in each eye that underwent PK. The extent of ocular involvement was graded according to published criteria [13, 14]. Mild disease was defined by the presence of corneal opacity along with a normal iris and lens. Moderate disease was defined by the presence of adherent iris strands or other iris defects such as atrophy or coloboma. Severe disease was defined by the presence of keratolenticular adhesion or cataract. As part of systemic work-ups, patients were referred to a pediatrician to identify any systemic anomalies. 

### Surgical procedures and postoperative care

All keratoplasties were performed under general anesthesia by one surgeon (JJX), with a standard technique as previously described [15]. The graft size was 6.0 to 7.5 mm in diameter. Overall, a graft-host disparity of 0.25–1.0 mm was maintained. The majority of the grafts (*n* = 62, 87.3%) were 0.5 mm larger than the host cornea bed. Synechiolysis was performed when iridocorneal adhesion (ICA) was observed. Lens surgery, combined with anterior vitrectomy, was performed if cataract or corneal lenticular touch was identified. Excised host corneas were sent for histopathological examination.

Routine postoperative medications included topical corticosteroids, topical antibiotics, and a topical immunosuppressant (tacrolimus 0.05% eye drops). Topical antiglaucoma medications were prescribed when IOP increased over 21 mmHg by repeated measurements. Suture removal was performed under general anesthesia when suture loosening or exposure was observed, as soon as wound healing permitted. The median time interval from corneal transplantation to suture removal was 2 months (mean: 2.7 months, range: 0.5–12 months).

### Imaging instrumentation

Anterior segment OCT scans were performed under general anesthesia, using a portable spectral-domain OCT system (iVue 100–2; Optovue, Fremont, California, USA). A Pachymetry + Cpwr scan pattern (6 mm scan diameter, eight radials, 1024 axial scans each, repeated five times) was used to map the central cornea over a 6 mm diameter area. A crossline scan mode (scanning width of 6 mm, axial resolution of 5 μm) was adopted to visualize the peripheral cornea, the graft-host interface, and the anterior chamber angle at an interval of 45°. Preoperative OCT images were acquired before corneal transplantation. During follow-up, infants and children underwent postoperative OCT examinations mainly at the time of suture removal. Two experienced observers (JX and YY) analyzed the OCT images together and recorded the extent of ICA and anterior chamber depth (ACD), by using computer calliper tools installed with the OCT device.

### Statistical analysis

Data analysis was performed using SPSS V.21.0 software (SPSS Inc., Chicago, Illinois, USA). Basic descriptive statistics were calculated based on all data gathered, and values were reported as means ± standard deviation. For comparative purposes, a Mann–Whitney U test was used for continuous parameters, and a chi-square test was used for categorical variables. A stepwise logistic regression analysis was performed to assess potential risk factors for postoperative AS. All the tests were two-tailed, and a *p*-value < 0.05 was considered statistically significant.

## Results

### Ophthalmic and systemic features

A total of 71 PKs were performed in 58 patients (30 males and 28 females) diagnosed with Peters’ anomaly. Of these patients, 38 had bilateral corneal opacity and 13 underwent bilateral grafting. Four patients (6.9%) had associated systemic anomalies including cardiopulmonary anomaly (*n* = 3) and developmental delay (*n* = 1). In those 71 eyes that underwent PK, 6 (8.4%) were classified as having mild disease, 58 (81.7%) had moderate disease, and 7 (9.9%) had severe disease. The average age at the time of surgery was 9.6 ± 4.1 months (range, 5–23 months, median: 8 months). Combined cataract surgery was performed in 3 eyes. The average preoperative IOP was 17.9 ± 6.7 mmHg in all affected eyes. Corneal vascularization was observed in 43 eyes (60.6%). The horizontal and the vertical corneal diameters of the affected eyes were measured as 9.6 ± 0.8 mm and 8.9 ± 0.8 mm, respectively. Using anterior segment OCT, we observed various degrees of preexisting ICA, and shallow anterior chamber was detected. The mean value of ACD was measured to be 1.2 ± 0.4 mm, which was relatively smaller as compared with that in normal infants (3.1 mm) [16]. Detailed clinical features are summarized in Table 1.

**Table 1:** Clinical findings of patients with Peters’ anomaly before and after penetrating keratoplasty

Variables	Total (*n* = 71)	Mild (*n* = 6)	Moderate (*n* = 58)	Severe (*n* = 7)
Age (month)	9.6 ± 4.1	8.5 ± 2.4	9.5 ± 3.9	11.3 ± 6.3
Preoperative IOP (mmHg)	17.9 ± 6.7	15.3 ± 4.2	18.2 ± 7.2	17.3 ± 2.8
Anterior chamber depth (mm)	1.20 ± 0.41	1.34 ± 0.22	1.27 ± 0.36	0.53 ± 0.29
Preexisting ICA				
None	7 (9.9%)	6 (100%)	0	1 (14.3%)
1,2 quadrants	26 (36.6%)	0	26 (44.8%)	0
3,4 quadrants	38 (53.5%)	0	32 (55.2%)	6 (85.7%)
Corneal diameter (mm)				
Horizontal	9.6 ± 0.8	10.1 ± 0.7	9.6 ± 0.8	8.9 ± 0.8
Vertical	8.9 ± 0.8	9.8 ± 0.8	8.9 ± 0.8	8.5 ± 0.8
Graft size (mm)	6.6 ± 0.3	6.8 ± 0.4	6.6 ± 0.3	6.4 ± 0.2
Corneal vascularization				
None	28 (39.4%)	1 (16.7%)	24 (41.4%)	3 (42.9%)
Associated	43 (60.6%)	5 (83.3%)	34 (58.6%)	4 (57.1%)
Surgery				
PK	68 (95.8%)	6 (100%)	58 (100%)	4 (57.1%)
PK + ECCE	3 (4.2%)	0	0	3 (42.9%)
Postoperative anterior synechiae				
None	12 (16.9%)	4 (66.7%)	8 (13.8%)	0
1,2 quadrants	36 (50.7%)	2 (33.3%)	31 (53.4%)	3 (42.9%)
3,4 quadrants	23 (32.4%)	0	19 (32.8%)	4 (57.1%)
Follow-up per eye (months)				
mean	8.8 ± 6.5	8.5 ± 6.0	9.0 ± 6.6	7.3 ± 6.4
median	7.0	7.3	7.0	6.0
range	1- 28	2.5–18	1–28	1.3–18
Clear graft	60 (84.5%)	6 (100%)	50 (86.2%)	4 (57.1%)

*IOP* intraocular pressure, *ICA* iridocorneal adhesion, *PK* penetrating keratoplasty, *ECCE* extracapsular cataract extraction

### Results of penetrating keratoplasty

Patients were followed from the time of PK for a mean of 8.8 ± 6.5 months (range, 1–28 months). Clear grafts at the most recent follow-up were obtained in 84.5% of transplanted eyes, eleven of 71 eyes had cloudy grafts. At final follow-up, the mean postoperative IOP was 14.9 ± 4.0 mmHg. Twenty-seven eyes of 23 patients presented with IOP elevation and received topical antiglaucoma drug therapy, with a median of 2 (interquartile range, 1–2) IOP-lowering medications. Other postoperative complications included rejection (10 eyes), cataract (6 eyes), persistent epithelial defect (4 eyes), wound dehiscence (2 eyes), and infection (1 eye).

### Postoperative anterior synechiae

Postoperative OCT scans showed AS formation in 59 eyes (83.1%). Table 2 compares the baseline characteristics of eyes with and without postoperative AS. The results showed that disease severity (*p* = 0.002), ACD (*p* = 0.003), corneal diameter (*p* = 0.009), graft size (*p* = 0.001), and preexisting ICA (*p* = 0.003) were significantly different between the two groups. Univariate logistic regression analysis also demonstrated that these covariates had statistically significant associations with postoperative AS (Table 3). In the multivariable model, small graft size (odds ratio [OR] = 0.016, 95% confidence interval [CI] 0.000–0.529, *p* = 0.020), preexisting ICA (OR = 16.639, 95% CI 1.494–185.294, *p* = 0.022), and small ACD (OR = 0.009, 95% CI 0.000–0.360, *p* = 0.012) were found to be independent risk factors for postoperative AS (Table 3).

**Table 2:** Baseline characteristics of eyes undergoing penetrating keratoplasty with and without postoperative anterior synechiae

Variables	Postoperative AS ( +)	Postoperative AS (-)	*p*-value
Age (months)	9.5 ± 4.0	10.2 ± 4.8	0.424^*^
Sex			0.366^†^
Male	33 (86.8%)	5 (13.2%)	
Female	26 (78.8%)	7 (21.2%)	
Severity			**0.002**^†^
Mild	2 (33.3%)	4 (66.7%)	
Moderate	50 (86.2%)	8 (13.8%)	
Severe	7 (100%)	0	
ACD (mm)	1.14 ± 0.42	1.50 ± 0.23	0.003^*^
Corneal diameter (mm)	9.5 ± 0.8	10.1 ± 0.6	0.009^*^
Graft size (mm)	6.5 ± 0.3	6.9 ± 0.3	0.001^*^
Graft-host disparity (mm)	0.53 ± 0.12	0.48 ± 0.07	0.180^*^
Width of host corneal bed (mm)	2.9 ± 0.7	3.2 ± 0.5	0.107^*^
Preexisting ICA			**0.003**^†^
Yes	56 (87.5%)	8 (12.5%)	
No	3 (42.9%)	4 (57.1%)	
Internal GHJ Alignment^a^			** < 0.001**^†^
Well-apposed	56 (24.8%)	170 (75.2%)	
Malapposed	150 (43.9%)	192 (56.1%)	
Lens abnormality			0.209^†^
Yes	7 (100%)	0	
No	52 (81.3%)	12 (18.7%)	
Stromal vessels			0.411^†^
Yes	37 (86.0%)	6 (14.0%)	
No	22 (78.6%)	6 (21.4%)	

^*^ *P* values are based on Mann–Whitney U test; *p* < 0.05 considered statistically significant

^†^ *P* values are based on chi-square test; *p* < 0.05 considered statistically significant

^a^ Excluded were the data from eyes without GHJ synechiae

*AS* anterior synechiae, *ACD* anterior chamber depth, *ICA* iridocorneal adhesion, *GHJ* graft-host junction

**Table 3:** Associations between baseline covariates and postoperative anterior synechiae formation

Variables	Univariable Model			Multivariable Model		
	**Regression Coeffient**	**OR (95%CI)**	*p*-value*	**Regression Coeffient**	**OR (95%CI)**	*p*-value*
Age	-0.038	0.963 (0.833, 1.112)	0.604	—	—	—
Sex (female vs male)	-0.575	0.563 (0.160, 1.979)	0.370	—	—	—
Corneal diameter	-0.999	0.368 (0.158, 0.860)	**0.021**	0.555	1.743 (0.376, 8.075)	0.478
Graft size	-3.156	0.043 (0.005, 0.366)	**0.004**	-4.126	0.016 (0.000, 0.529)	**0.020**
Width of host corneal bed	-0.621	0.537 (0.217, 1.332)	0.180	—	—	—
Preexisting ICA (yes vs no)	2.234	9.333 (1.757, 49.591)	**0.009**	2.812	16.639 (1.494, 185.294)	**0.022**
ACD	-3.058	0.047 (0.005, 0.471)	**0.009**	-4.732	0.009 (0.000, 0.360)	**0.012**
Stromal vessels (yes vs no)	0.520	1.682 (0.483, 5.862)	0.414	—	—	—
Suture retention time	-0.136	0.873 (0.669, 1.140)	0.320	—	—	—

^*^P values are based on logistic regression analysis; *p* < 0.05 considered statistically significant

*ICA* iridocorneal adhesion, *ACD* anterior chamber depth, OR odds ratio, *CI* confidence interval

**Fig. 1:**
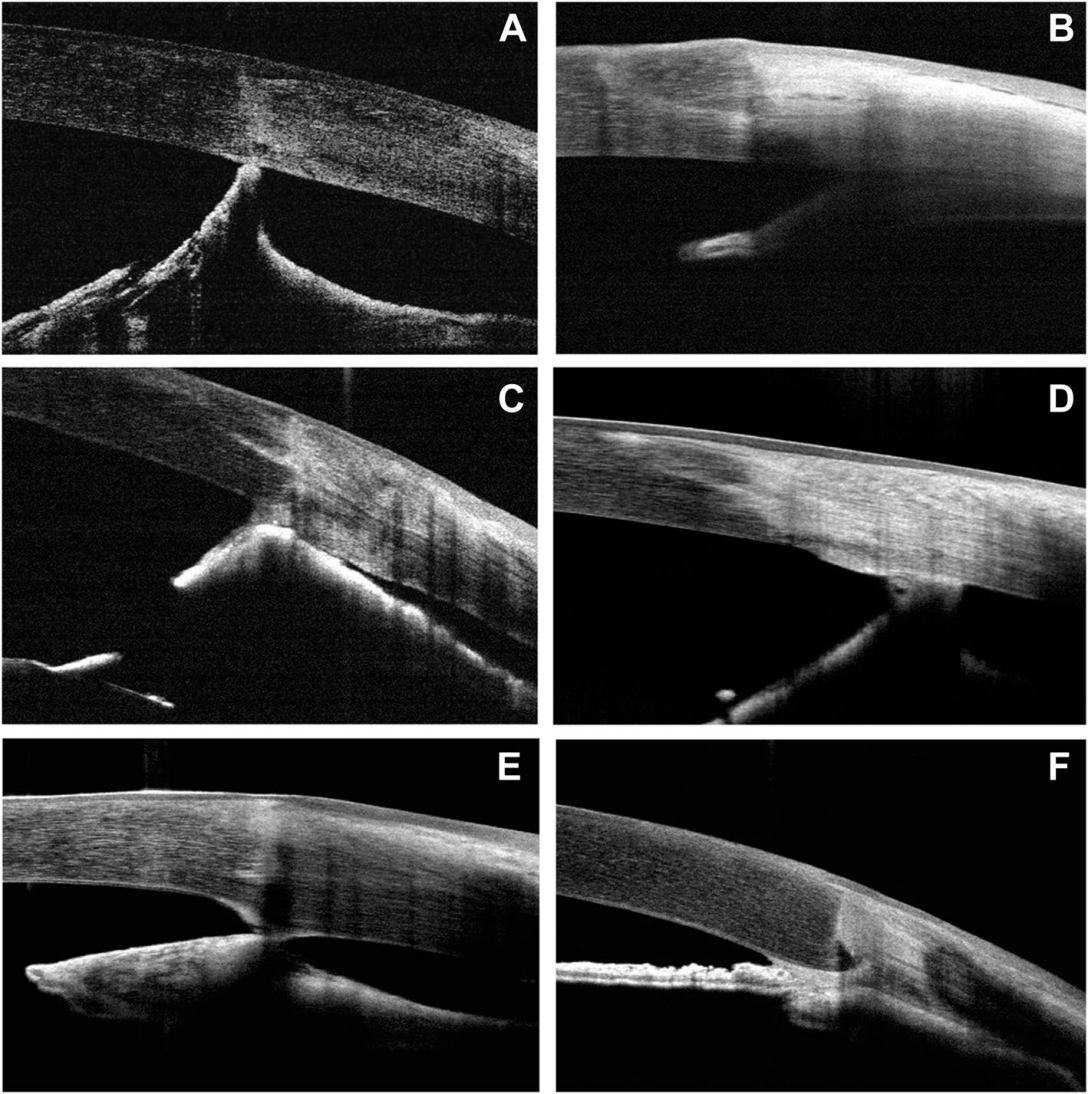
Types of anterior synechiae following penetrating keratoplasty in cases of Peters’ anomaly. Anterior segment optical coherence tomography images showed graft-host junction synechiae (**A**, **C**, **E**), peripheral synechiae (**B**, **D**), and a combination of both (**F**)

**Fig. 2:**
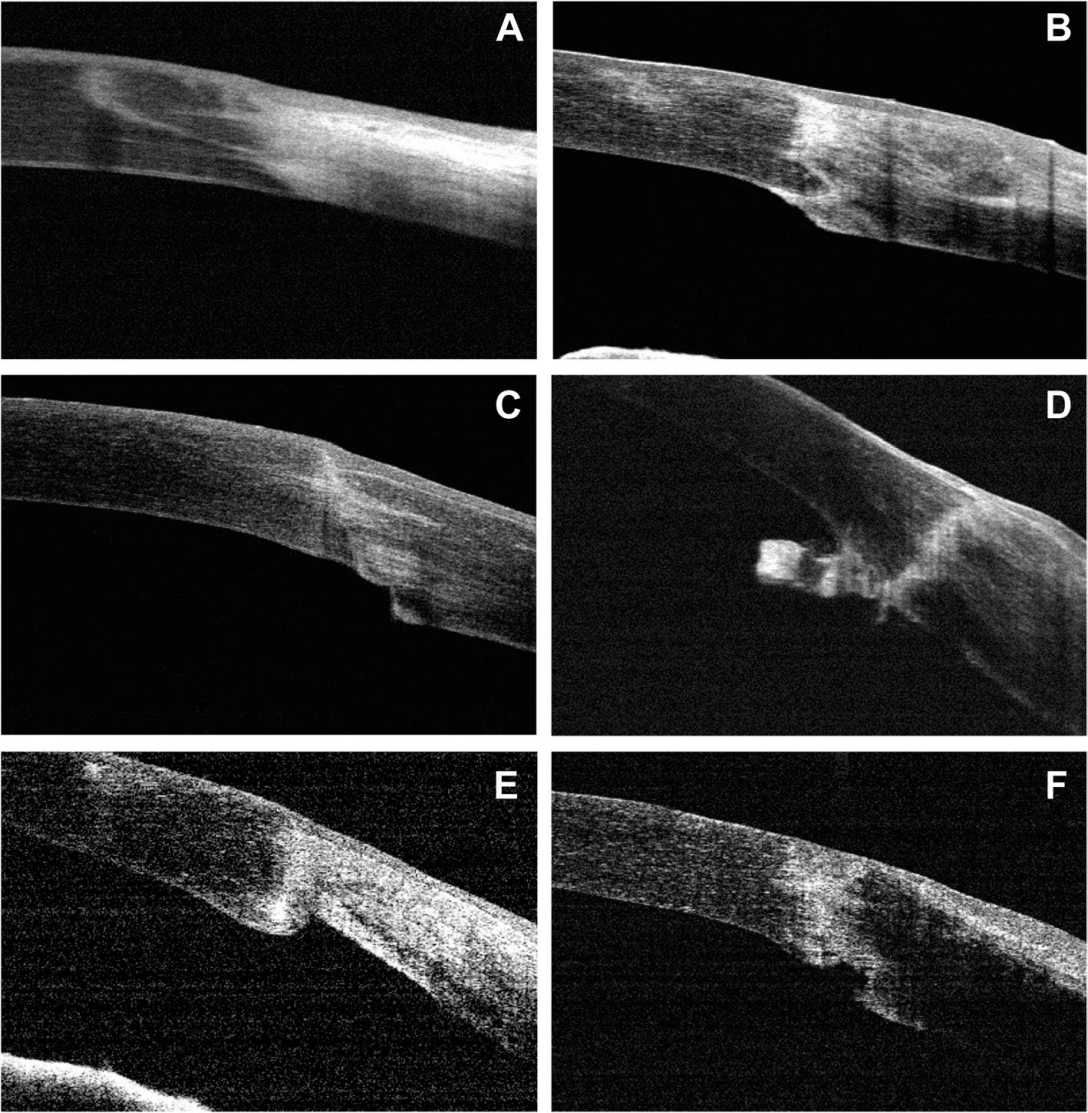
Main types of malpositions found in the internal graft-host junctions following penetrating keratoplasty in Peters’ anomaly. In addition to well-apposed junctions (**A**), we also noted steps, either host steps (**C**) or graft steps (**E**), protrusions such as hills (**B**) or tags (**D**), and gapes (**F**)

The types of AS noted on the OCT images included synechiae at the graft-host junction (GHJ) in 30 (50.8%) eyes, synechiae at the peripheral host cornea in 7 (11.9%) eyes, and a combination of both sites in 22 (37.3%) eyes (Fig. 1). A total of 568 GHJ sections were analyzed. All the GHJs had a continuous smooth epithelial surface, while 342 of them (60.2%) showed malpositions of the internal surface in the forms of gapes, steps, and protrusions (Fig. 2), as previously described by Kaiserman et al. [17]. GHJ synechiae were presented in 150 (43.9%) of the malapposed junctions, and 56 (24.8%) of the well-apposed junctions. A chi-square test showed a significant difference (*p* < 0.001) between the two groups (Table 2), suggesting that the internal malapposition of the corneal wound was associated with AS formation.

**Fig. 3:**
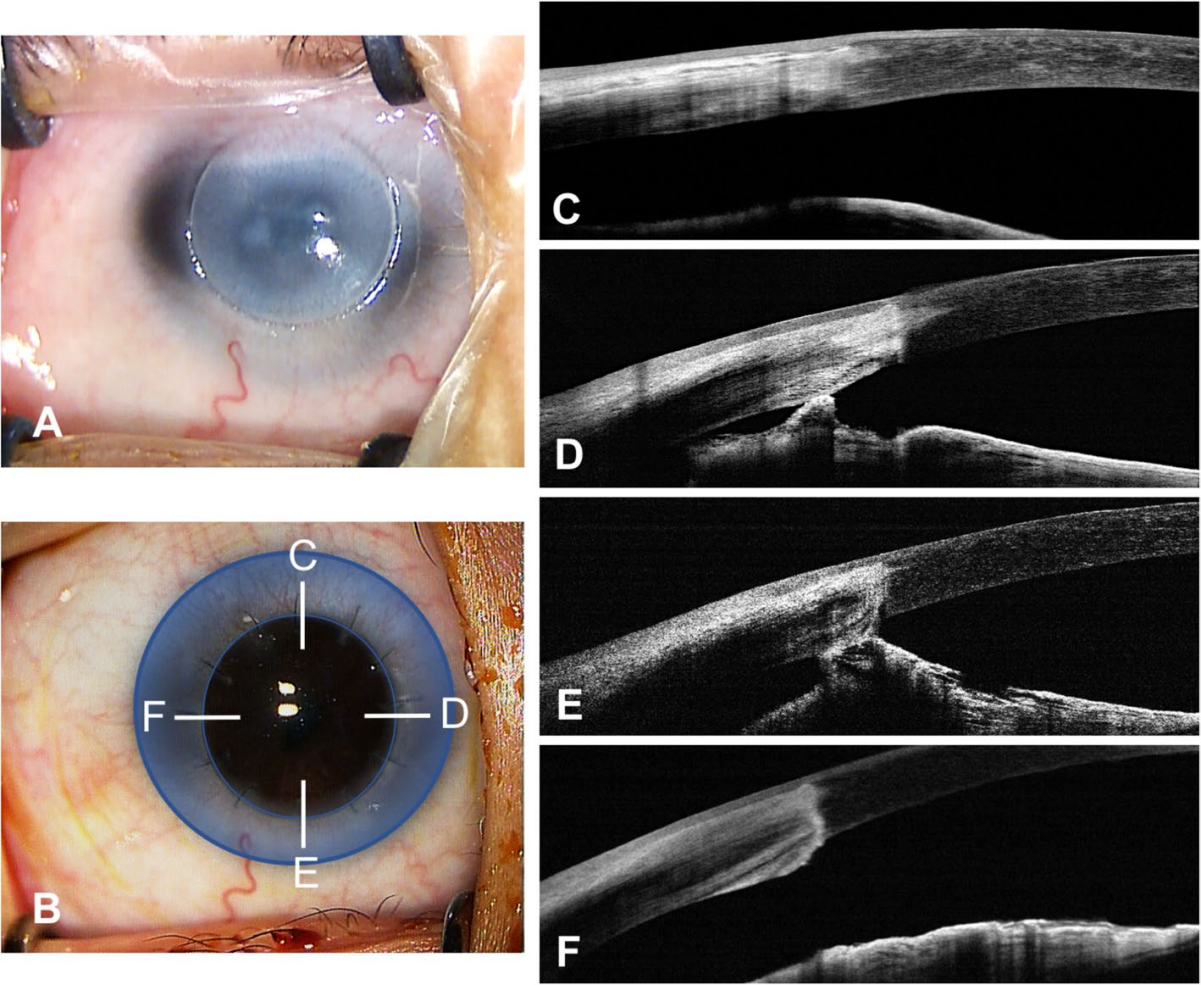
A 10-month-old boy with moderate Peters’ anomaly had penetrating keratoplasty in his right eye. **A** A surgical microscope image of the patient’s cornea is shown, with the opaque cornea trephined. **B** Three months postoperatively, the graft was well-centered and clear. The blue area shows the host corneal bed. Anterior segment optical coherence tomography images show the formation of postoperative anterior synechiae in the nasal (**D**) and the inferior (**E**) quadrants, while no iridocorneal adhesion was observed in the superior (**C**) or the temporal (**F**) quadrants

Among the eyes presented with postoperative AS, 36 had synechiae in one or two quadrants, and 23 had three or four quadrants involved. Findings from the logistic regression analysis identified that larger areas of preexisting ICA (OR = 9.609, 95% CI 1.872–49.329, *p* = 0.007) and the width of the host corneal bed (OR = 0.311, 95% CI 0.109–0.885, *p* = 0.029) were independent risk factors for increased postoperative AS (Table 4). Here we defined the host corneal bed as the residual part of the recipient cornea after trephination (Fig. 3). Eyes with grafts closer to the corneal limbus had narrower host corneal beds.

**Table 4:** Associations between baseline covariates and postoperative anterior synechiae > 2 quadrants

Variables	Univariable model			Multivariable model		
	**Regression Coeffient**	**OR (95%CI)**	*p*-value*	**Regression Coeffient**	**OR (95%CI)**	*p*-value*
Age	-0.008	0.992 (0.868, 1.134)	0.905	—	—	—
Sex (female vs male)	-0.331	0.718 (0.248, 2.079)	0.542	—	—	—
Corneal diameter	-1.190	0.304 (0.126, 0.735)	**0.008**	—	—	—
Graft size	-0.658	0.518 (0.077, 3.490)	0.499	—	—	—
Width of host corneal bed	-1.341	0.262 (0.098, 0.701)	**0.008**	-1.170	0.311 (0.109, 0.885)	**0.029**
Preexisting ICA > 2 quadrants (yes vs no)	2.463	11.735 (2.390, 57.614)	**0.002**	2.263	9.609 (1.872, 49.329)	**0.007**
ACD	-1.083	0.339 (0.090, 1.279)	0.110	—	—	—
Abnormal lens (yes vs no)	0.840	2.316 (0.468, 11.468)	0.304	—	—	—
Stromal vessels (yes vs no)	0.818	2.267 (0.725, 7.084)	0.159	—	—	—
Suture retention time	-0.089	0.915 (0.687, 1.219)	0.545	—	—	—

^*^P values are based on logistic regression analysis; *p* < 0.05 considered statistically significant

*ICA* iridocorneal adhesion, *ACD* anterior chamber depth, *OR* odds ratio, *CI* confidence interval

## Discussion

Anterior segment OCT has proven a valuable technique in the clinical assessment of children with congenital corneal opacity [11]. High-resolution cross-sectional images obtained on OCT prior to surgery supplemented routine ophthalmic evaluation and served as a useful diagnostic tool for appropriate surgical planning. Our previous study described the in vivo OCT features of infants with Peters’ anomaly and demonstrated that this modality aided in better tissue handling by effectively detecting areas of pre-existing iris/lens adhesions and facilitated appropriate concomitant procedures such as cataract extraction and synechiolysis [13]. During surgery, screening pediatric corneal opacities with intraoperative OCT facilitates minimally invasive surgical approaches by providing real-time dynamic feedback of the tissue alterations, and guides enhanced decision making [12]. Intraoperative OCT has already been described in corneal transplantation, especially for lamellar keratoplasty such as DMEK and DSAEK [18–20].

In this study, we performed OCT to observe the anterior segment structures and to evaluate anterior synechiae following PK in patients with Peters’ anomaly. We found that 83.1% of the operated eyes had postoperative AS, which varied in range and site. Over half of the cases developed synechiae within two quadrants. The adherent iris strands were most seen at the site of the internal GHJs, other sites included the peripheral host cornea with or without angle synechiae.

AS is a frequent complication encountered after PK. Peripheral AS may cause secondary angle-closure glaucoma and lead to graft failure. AS at the graft-host interface was regarded as a poor prognostic factor, associated with the loss of graft clarity via interfering with the adequate wound healing of the posterior aspect of the cornea and promoting endothelial disturbances, retrocorneal membranes, and corneal vascularization, which facilitated immune rejection [21, 22]. The reported incidence of peripheral AS after PK in adult patients varied between 8% and 46.7%, with or without IOP elevation [23–25]. Our results revealed a considerably higher prevalence rate of postoperative AS in infants and children with Peters’ anomaly.

Pediatric PK is more complicated and technically more difficult than adult PK due to decreased scleral rigidity, alarming posterior pressure, anterior movement of the lens-iris diaphragm, and increased fibrin release, which may lead to the adherence of the iris to the peripheral cornea and the wound. The present study found that preexisting iris-corneal adhesion and a shallow anterior chamber were risk factors for postoperative synechiae. Corneal opacification, along with iridocorneal adhesions, is one of the major characteristics of Peters’ anomaly. In addition, such patients have smaller anterior segment dimensions. During surgery, they are more likely to experience hemorrhage and injury to the iris, which may intensify the inflammatory response and fibrinous reaction, causing postoperative AS. On the other hand, it is hypothesized that due to long-standing adhesion, the iris may become flaccid with advanced atrophic changes and tends to retain this abnormal position, leading to the reformation of synechiae postoperatively [26]. Because patients with severe disease often had larger areas of preexisting iris-corneal touch and smaller ACD values [13], they were found to be more susceptible to generating postoperative AS.

Preexisting iridocorneal adhesion has been identified as a poor prognostic factor. Yang et al. reported that the graft failure rate was higher in eyes with preexisting iridocorneal adhesions over two quadrants in cases of Peters’ anomaly [14]. Other studies have found that preoperative diagnoses, such as adherent leukoma and infectious keratitis, featured with preexisting iridocorneal adhesions and inflamed floppy iris, increased the odds of postoperative AS and secondary angle-closure glaucoma [9, 27, 28]. It is paramount to perform synechiolysis after the wound is closed to help prevent postoperative AS formation. Despite optimal synechiolysis, a high prevalence rate of postoperative AS was still observed in the current study.

Other factors contributing to the formation of postoperative AS in these patients included small corneal size and small graft size. In eyes with Peters’ anomaly, the size of the donor tissue was tailored to the size of the opacity and of the host cornea. Because of the increased elasticity of the infant cornea and sclera, it is recommended that the diameter of the donor tissue be 0.5–1.0 mm larger than the recipient opening [29]. In the current study, 95.8% of the cases had a graft-host disparity within this range. Also, there was a significant correlation between the graft size and the recipient corneal size (*p* = 0.005). Eyes grafted with larger donor cornea buttons may have a larger corneal size and thus allow adequate space for the anterior segment during the process of wound healing and wound retraction, and may help reduce the risk of postoperative synechiae. Moreover, a negative correlation was identified between the width of the host corneal bed and quadrants of postoperative AS. This suggested that the narrower the host corneal bed was, the closer the surgical manipulation and the GHJs were to the corneal limbus, which may induce larger amounts of AS.

In this study, an association between malapposition of the internal GHJ and postoperative AS was also observed. The prevalence of GHJ malpositions in the patient population was 60.2%, which was similar to that previously reported in cases of adult PK [17, 30]. AS formation might follow such malposition because the iris tends to adhere to bare stroma more readily [31]. Proper wound apposition and meticulous wound closure are essential to avoid iris incarceration in the wound intraoperatively and may prevent or lessen the risk of postoperative synechiae [25, 31]. However, achieving proper tissue apposition and wound closure can be difficult due to thinner and more pliable corneal tissue, anterior displacement of the lens-iris diaphragm, and wound retraction. Graft-host misalignment may be induced and increase the chances of adherence of the iris to the surgical wound.

The limitations of this study are mainly related to its short-term nature. In 27 eyes (38.0%), the most recent follow-up information was obtained within 6 months after PK. Postoperative OCT examinations were performed mainly at the time of suture removal, due to the requirement of general anesthesia. After the sutures were completely removed, we lost track of the OCT evaluations on many young patients. In cases where AS formation is a concern, the operated eyes should be closely watched for the development of glaucoma, graft rejection, and graft survival. Hence, a hand-held OCT device is recommended in serial follow-ups to observe AS progression, assess details of the anterior segment, and clarify the consequences of postoperative AS in this specific population. Second, with the current OCT system (scanning wavelength = 830 nm), the presence of thick and dense opacity on the remaining cornea could impede optimal imaging of the peripheral anterior chamber. In that case, synechiae could not be precisely localized and synechiolysis might be incomplete. A longer-wavelength OCT modality that allows for higher penetration and reproducible measurements may be useful and complementary in postoperative management. Third, this preliminary study remained incomplete regarding surgical and visual outcomes. Further analysis is ongoing for the association between long-term prognosis and OCT findings.

Although PK remains the dominant surgical approach to clear the visual axis in patients with Peters’ anomaly, it is still considered a high-risk procedure associated with both intraoperative and postoperative complications. Clinicians have been seeking alternatives to treat these patients with minimal invasion, fewer complications, and better outcomes. Hashemi et al. reported 2 cases of DSAEK in type II Peters’ anomaly [32]. Both patients had improved vision, and a reduction in the density of opacity and retained a clear graft up to 1-year after the operation. Because this disease mostly affects the posterior layers of the cornea, DSAEK may be a viable option in properly selected cases (those with a more prominent and localized posterior corneal opacity and a minimum anterior corneal involvement). DSAEK offers advantages over PK in terms of less intraoperative complications related to an open globe, decreased risk of suture-related complications, quicker visual rehabilitation, and better refractive outcomes. However, challenges and some shortcomings to this technique should be noted. DSAEK in children with Peters’ anomaly is technically more difficult in scoring the Descemet membrane due to stronger adhesion between the Descemet membrane and stroma, poor visualization through the overlying opacified cornea, and the shallower anterior chamber. DSAEK also may not provide as clear a cornea as PK, some residual haze and the overall thick cornea may remain after the procedure, precluding adequate visualization. With limited experience, the role of DSAEK in the management of Peters’ anomaly remains inexplicit. Further studies are needed to define the safety and to compare the outcomes with that of conventional PK in a long-term follow-up.

In conclusion, this retrospective study provides insights into the characteristics of anterior synechiae after PK in pediatric patients with Peters’ anomaly. We demonstrated that AS was a common postoperative complication in this specific population and several risk factors were associated with its occurrence. The results allow predictions to be made regarding which patients are more likely to form postoperative AS and may help clinicians to manage this rare yet challenging disease. Still, the association between postoperative AS and clinical outcomes of PK (graft survival, visual gain) remains to be determined. Anterior segment OCT can be considered as a useful imaging technique for clinical evaluation and surgical follow-up of patients with Peters’ anomaly. The development of handheld devices, scanning with a longer wavelength, is expected to improve the standard of medical care.

## Supplementary Information

**Additional file 1: Table S1.** Differences in anterior synechiae (AS) between type 1 and type 2 Peters’ anomaly.

## Data Availability

The data that support the findings of this study are available on request from the corresponding author JJX. The data are not publicly available because part of them contained information that could compromise research patient privacy.

## Abbreviations

AS: Anterior synechiae
PK: Penetrating keratoplasty
OCT: Optical coherence tomography
GHJ: Graft-host junction
ICA: Iridocorneal adhesion
OR: Odds ratio
CI: Confidence interval
IOP: Intraocular pressure
ACD: Anterior chamber depth
DMEK: Descemet’s membrane endothelial keratoplasty
DSAEK: Descemet’s stripping automated endothelial keratoplasty
