# Supplementary material for: Anterior synechiae after penetrating keratoplasty in infants and children with Peters’ anomaly
Source: BMC Ophthalmol. 2022 Jun 9;22:259. doi: 10.1186/s12886-022-02473-0 (PMC9178809; doi:10.1186/s12886-022-02473-0)
Supplement: Supplementary file 1 — Additional file 1: Table S1. Differences in anterior synechiae (AS) between type 1 and type 2 Peters’ anomaly. [file 12886_2022_2473_MOESM1_ESM.docx]

|  |  | Type 1 (n=64) | Type 2 (n=7) | Total (n=71) |
| --- | --- | --- | --- | --- |
| postoperative AS (-) | | 12 (18.8%) | 0 | 12 (16.9%) |
| postoperative AS (+) | | 52 (81.2%) | 7 (100%) | 59 (83.1%) |
| AS site | GHJ | 26 (40.6%) | 4 (57.1%) | 30 (42.3%) |
|  | peripheral host cornea | 7 (10.9%) | 0 | 7 (9.8%) |
|  | combined | 19 (29.7%) | 3 (42.9%) | 22 (31.0%) |
| AS range | 1~2 quadrants | 33 (51.5%) | 3 (42.9%) | 36 (50.7%) |
|  | 3~4 quadrants | 19 (29.7%) | 4 (57.1%) | 23 (32.4%) |

**Table S1. Differences in anterior synechiae (AS) between type 1 and type 2 Peters’ anomaly**

Legend：

Type 1 Peters’ anomaly (64 eyes) consisted of the mild and moderate cases, the more severe forms with lens abnormalities corresponded to type 2 disease (7 eyes). Postoperative AS was observed in 81.2% (52/64) of type 1 patients and in all of type 2 patients. Compared with type 1, type 2 tended to have more diffuse and more extensive AS following penetrating keratoplasty.
